# Methotrexate showed efficacy both in Crohn’s disease and ulcerative colitis, predictors of surgery were identified in patients initially treated with methotrexate monotherapy

**DOI:** 10.3389/fphar.2022.996065

**Published:** 2022-09-26

**Authors:** Mengyao Wang, Jingwen Zhao, Heran Wang, Changqing Zheng, Bing Chang, Lixuan Sang

**Affiliations:** ^1^ Department of Gastroenterology, Shengjing Hospital of China Medical University, Shenyang, China; ^2^ Department of Gastroenterology, The First Affiliated Hospital of China Medical University, Shenyang, China

**Keywords:** inflammatory bowel disease, methotrexate monotherapy, prognostic analysis, Crohn’s disease, ulcerative colitis

## Abstract

**Objective:** This study aimed to evaluate methotrexate efficacy in patients with Crohn’s disease (CD) and ulcerative colitis (UC), and identify predictors of surgery for patients who were initially treated with methotrexate monotherapy.

**Design:** We performed a retrospective analysis of 34,860 patients with inflammatory bowel disease (IBD) in the IBD Bioresource (United Kingdom) prior to 9 November 2021. Logistic regression was used to identify factors associated with methotrexate efficacy. The data were randomly stratified into training and testing sets (7:3). Nomograms were developed based on Cox regression analysis outcomes. The predictive accuracy and discriminative ability were determined using the concordance index (C-index) and calibration curves.

**Results:** Overall, 1,042 patients (CD: 791, UC: 251) were included. Independent factors associated with effective methotrexate monotherapy were younger age at diagnosis, latest therapy period, exclusive upper gastrointestinal tract disease (for CD), and longer duration between diagnosis and methotrexate initiation (for UC). For CD, predictors in the nomogram were gender, treatment era, tolerance, lesion site, perianal involvement, disease behaviour, and biologics requirements (C-index: 0.711 and 0.732 for training and validation cohorts, respectively). For UC, the factors were age at diagnosis and sex (C-index: 0.784 and 0.690 for training and validation cohorts, respectively). Calibration curves demonstrated good agreement between predictions and actual observations.

## Introduction

Inflammatory bowel diseases (IBD), including ulcerative colitis (UC), Crohn’s disease (CD), and IBD-unclassified (IBDU), are disorders of chronic intestinal inflammation characterised by repeated aggravation and remission. Its increasing incidence has caused a huge economic burden on patients and country’s healthcare systems (Kaplan, 2015; Rosen et al., 2015; Ng et al., 2017; Coward et al., 2019). Conventional immunomodulators, including methotrexate and thiopurines, have been the mainstay of IBD treatment for decades, especially to maintain remission in steroid-dependent or steroid-refractory patients (Sales-Campos et al., 2015; Torres et al., 2020a; Ran et al., 2021; Raine et al., 2022).

Methotrexate, which was initially developed in 1948 for the treatment of leukemia (Gabbani et al., 2016), has been clinically used for other diseases, including IBD, psoriasis, and rheumatoid arthritis (Tung and Maibach, 1990; Rajitha et al., 2017). Due to hepatotoxicity and gastrointestinal symptoms, patients treated with methotrexate should routinely undergo a full blood count and liver and pancreatic function tests during the treatment period (Cervoni et al., 2020; Vasudevan et al., 2020; Ran et al., 2021). In recent years, with the emergence of biological agents and concerns regarding their adverse effects, the application of methotrexate in IBD has been questioned.

The European Crohn’s and Colitis Organisation (ECCO) Guidelines on Therapeutics for patients with CD recommend parenteral administration of methotrexate for the maintenance of remission in steroid-dependent patients (Torres et al., 2020b). A multicentre randomised control trial (RCT) (Feagan et al., 1995) study of 141 patients with chronically active CD found that after 16 weeks, 37 patients (39.4%) in the methotrexate group were in clinical remission compared with 9 patients (19.1%) in clinical remission in the placebo group (*p* = 0.025), moreover the methotrexate group had received less prednisone than the placebo group (*p* = 0.025). However, there is not enough evidence supporting methotrexate for the maintenance of remission in UC patients. A French study (Carbonnel et al., 2016) assessed the efficacy of methotrexate in inducing UC steroid-free remission, after 16 weeks, methotrexate group was not superior to placebo with regards to induction of steroid-free remission (*p* = 0.15). Moreover, more patients in the placebo group experienced continued UC disease activity that required withdrawal from the aforementioned study. Further studies are needed to determine the effectiveness of methotrexate in UC maintenance therapy.

IBD has become a global problem characterised by a lifelong relapsing-remitting course, which diminishes the quality of life of patients and increases the consumption of healthcare resources. Thus, long-term efficacy is an important aspect of IBD treatment. A prior meta-analysis of 44 cohort studies suggested that for diagnosed patients, the 1-, 5- and 10- year surgery risk for patients with UC (colectomy with or without an ileal pouch–anal anastomosis) was 2.8%, 7.0%, and 9.6%, respectively, and for patients with CD (intestinal resection) was 12.3%, 18.0%, and 26.2%, respectively (Tsai et al., 2021). Because of the overall burden of IBD and its multifactorial aetiology, efforts should be made to improve the medical management of these inflammatory conditions. Identifying factors associated with surgery can help optimise the treatment plan.

## Methods

Here, we analyses the effectiveness of methotrexate in IBD treatment using the United Kingdom IBD BioResource (www.ibdbioresource.nihr.ac.uk), which launched in 2016 as part of the United Kingdom National Institute for Health Research BioResource (Parkes, 2019). The database contained a large number of patients whose information were ascertained at enrolment and updated annually, including demographic, clinical characteristics, and treatment informations. All patients signed consent forms allowing their information to be used by investigators. To obtain these data, a data appliycation was submitted to IBD BioResource.

### Patient population

A total of 34,860 patients in over 100 hospitals in the United Kingdom signed up before the data lock on 9 November 2021.

The following patients were excluded: 1) those whose basic information on IBD or treatment response was missing; 2) those who underwent surgery in the same year of methotrexate initiation; 3) those who started or were on biological therapy in the year of methotrexate initiation; and 4) those for whom we could not determine whether biological therapy or surgery overlapped the period of methotrexate monotherapy. Patients with UC who underwent colectomy prior to methotrexate initiation were also excluded. Surgery is common in CD progression; therefore, those who underwent surgery before methotrexate initiation were not excluded. IBDU was classified as UC in our study, since we were able to find UC-related information of IBDU patients in the database.

### Treatment effectiveness

Effectiveness was evaluated by asking the question, “Did methotrexate work?” Effectiveness was defined as answering “yes” and avoiding escalation to alternative (biologics and/or surgical) treatment during the methotrexate treatment. We also discussed the treatment duration between the initiation of methotrexate and escalation to biologics or surgery (whichever occurred first), which also reflected the effectiveness of methotrexate.

### Definitions in study

The diagnosis of IBD was made based on radiographic, endoscopic, histologic criteria, or physician judgment. For CD, disease location were classified as “ileal” (L1), “colonic” (L2), “ileo-colonic” (L3), “exclusive upper gastrointestinal tract (GI) Crohn’s” (L4); disease behaviour were classified as “stenosing”, “internal penetrating” and “inflammatory and others”; internal penetrating disease was defined if patients had evidence of entero-enteric or entero-vesicular fistulae, intra-abdominal abscesses or intestinal perforation; perianal involvement including tags, fissures, ulcers, perianal abscess, simple fistula (single fistula, little clinical problem), complex fistula (more than one or branching or recto-vaginal or major problem); surgery including colectomy, ileal or jejunal resection and the treatment of complications such as drainage of perianal abscess, perianal fistula repairment. For UC, disease location were divided into “procticis” (E1), “left sided” (E2), “extensive” (E3); surgery referred colectomy. Smoking history included current and past smoking. In Cox regression, biologics requirment referred to that patients requiring biologics after methotrexate initiation and before surgery; glucocorticoid requirment referred to that patients receiving intravenous glucocorticoids before or after methotrexate initiation, but before surgery.

### Statistical analysis

Continuous variables are presented as mean ± SD or median ± interquartile range (IQR), while categorical variables are presented as percentages or proportions. Continuous variables were analysed using Student’s t-tests or paired t-tests, as appropriate, while categorical variables were analysed using the chi-square or Fisher’s exact test. The Cochran-Mantel-Haenszel (CMH) test was applied to compare effectiveness of methotrexate between UC and CD, using stratified treatment initiation periods every 5 years. Multivariable logistic regression was performed to compare methotrexate effectiveness between patients with UC and CD, adjusting for age at diagnosis, gender, smoking history, treatment era, and time interval from diagnosis to methotrexate initiation. Next, univariate and multivariable logistic regression analyses were used to identify independent risk factors of methotrexate monotherapy effectiveness within UC and CD using the same covariates as above, but also including disease location (variables with *p* < 0.2 were included in multivariable regression). The CD and UC cohorts were randomly divided into training and validation cohorts at a ratio of 7:3. Univariate Cox regression was then used to screen for variables that were significantly correlated with surgery occurrence in the training group with the same covariates as above, but also included biologics, glucocorticoid treatment, and previous surgery. Predictors with *p* < 0.2 were fed into multivariable Cox regression model. Backward stepwise selection based on Akaike’s information criterion (AIC) was used to further eliminate redundant variables. The performance of the nomogram was evaluated using the concordance index (C-index) and a calibration curve in both the training and validation cohorts. A larger C-index indicates more accurate prognostic stratification. A *p*-value threshold of <0.05 was considered statistically significant in the adjusted models. All statistical analyses were performed using the R software (version 4.1.0).

## Results

### Patient population

**FIGURE 1:**
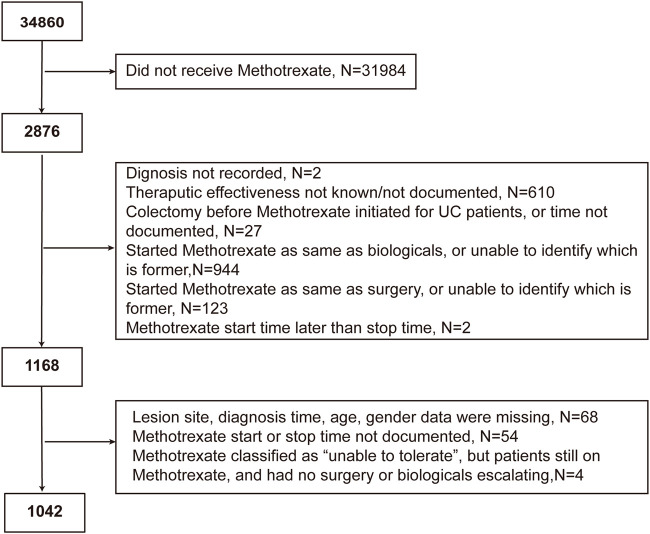
Flowchart of the study cohort depicting the inclusion and exclusion of patients from the analysis.

Of the 34,860 participants in the IBD BioResource at the data lock, 2876 (8.25%) had been treated with methotrexate (76.7% with CD, 21.7% with UC, and 1.6% with IBDU), either as monotherapy or combined with biologics. As shown in Figure 1, 1042 participants met the criteria for assessment of the effectiveness of methotrexate monotherapy [791 CD and 251 UC (including 15 IBDU cases)]. The baseline patient characteristics are presented in Table 1. The median age at CD and UC diagnosis was 30 and 38 years, respectively. Nausea was the most common adverse reaction in both patients with CD and UC. Patients who could not tolerate methotrexate were prone to have a nauseous reaction (CD: 30.2% vs. 10.1%, *p* < 0.001; UC: 31.8% vs. 6.1%, *p* < 0.001).

**TABLE 1:** Demographic and clinical characteristics and adverse reactions in 1042 patients who were treated with methotrexate monotherapy.

	CD (N = 791)			UC (N = 251)		
Age at diagnosis, median (IQR)	30.0 (20.0, 46.0)			38.0 (27.0, 49.0)		
Gender						
Male	330 (41.7%)			130 (51.8%)		
Female	461 (58.3%)			121 (48.2%)		
Smoke history						
No	481 (60.8%)			155 (61.8%)		
Yes	310 (39.2%)			96 (38.2%)		
CD Lesion						
Ileal (L1)	260 (32.9%)					
Colonic (L2)	232 (29.3%)					
Ileo-colonic (L3)	289 (36.5%)					
Exclusive upper GI Crohn’s (L4)	10 (1.3%)					
Perianal involvement	220 (27.8%)					
Behaviour						
Stenosing	224 (28.3%)					
Internal penetrating	70 (8.8%)					
Inflammatory and others	497 (62.8%)					
UC Lesion						
Procticis (E1)				22 (8.8%)		
Left sided (E2)				123 (49.0%)		
Extensive (E3)				106 (42.2%)		
Adverse reactions (χ2 test of tolerating and intolerating groups)	Tolerate (N = 632)	Unable to tolerate (N = 159)	*p* value	Tolerate (N = 229)	Unable to tolerate (N = 22)	*p* value
Abdominal pain and diarrhea	14 (2.2%)	10 (6.3%)	**0.016**	3 (1.3%)	1 (4.5%)	0.310
Blood cell decrease	6 (0.9%)	3 (1.9%)	0.390	1 (0.4%)	0 (0.0%)	1.000
Deranged LFTs	26 (4.1%)	16 (10.1%)	**0.005**	8 (3.5%)	1 (4.5%)	0.570
Flu-like symptoms	14 (2.2%)	10 (6.3%)	**0.016**	3 (1.3%)	1 (4.5%)	0.310
Nausea	64 (10.1%)	48 (30.2%)	**<0.001**	14 (6.1%)	7 (31.8%)	**<0.001**
Others	63 (10.0%)	50 (31.4%)	**<0.001**	35 (15.3%)	2 (9.1%)	0.750

Significant *p* values shown in bold; CD, Crohn’s disease; UC, ulcerative colitis; CI, confidence interval; GI, gastrointestinal tract; IQR, interquartile ranges.

**FIGURE 2:**
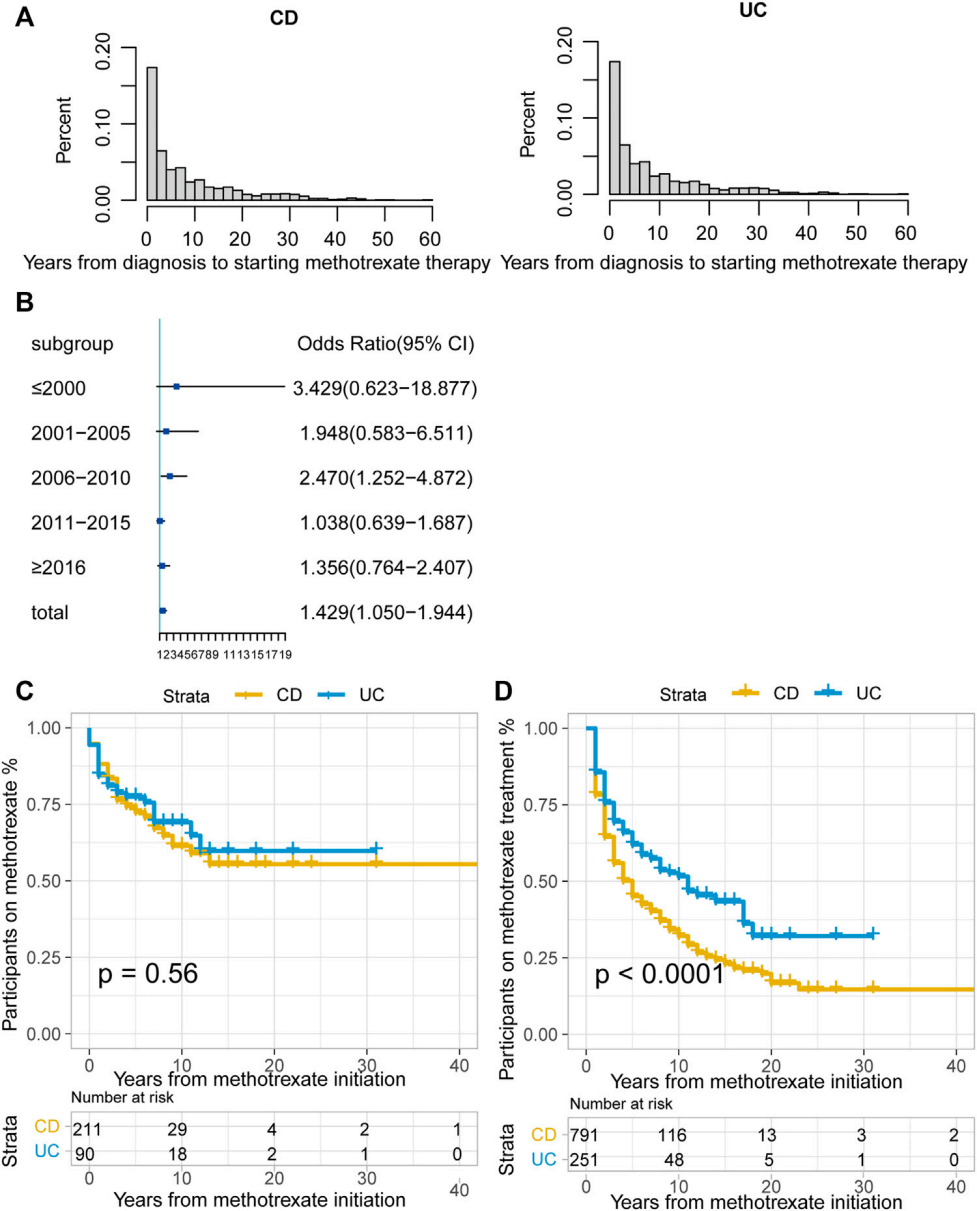
**(A)** Time interval (years) between CD or UC diagnosis and methotrexate monotherapy; **(B)** Odds ratio (OR) of methotrexate monotherapy being effective in UC vs. CD stratified by therapy period. The horizontal coordinates are OR values, blue boxes indicate OR values for every era, and black solid lines indicate 95% confidence intervals (CI) for OR values; **(C)** The Kaplan-Meier curve shows the duration of methotrexate treatment in patients in whom methotrexate monotherapy was considered effective; **(D)** The Kaplan-Meier curve shows the duration of effective methotrexate monotherapy without requirement for treatment escalation (*p* < 0.0001 for log-rank test). (CD, Crohn’s disease; UC, ulcerative colitis).

Among the 1042 patients, 181 (17.4%) were unable to tolerate and had to stop methotrexate. The median duration of methotrexate treatment was 2 years (IQR: <1–6 years) for patients with CD and 3 years (IQR: 1–7 years) for patients with UC. The median time from diagnosis to methotrexate initiation was 5 years in both patients with CD and UC (Figure 2A). A total of 486 (61.44%) patients with CD and 106 (42.2%) with UC received biologics after methotrexate monotherapy.

### Effectiveness of methotrexate monotherapy

Overall, 28.9% (301/1042) cases were regarded as effective, without escalation to biologics or surgery during treatment. Methotrexate monotherapy was effective in 35.5% of patients with UC (94/251) and a lower proportion of patients with CD (26.7%; 211/791, *p* = 0.001). In methotrexate-tolerant group, it was effective in 39.3% of patients with UC and 32.8% of patients with CD (*p* = 0.074).

According to the CMH test stratified by the treatment initiation era (every 5 years), methotrexate appeared to be more effective in UC than in CD (odds ratio [OR]: 1.429, 95% confidence interval [CI]: 1.050–1.944, *p* = 0.023). The test of homogeneity of ORs indicated that there was no heterogeneity in the OR values of each therapy area (*p* = 0.237). We used the forest map to display the OR values of each era, and the results were meaningful only for the 2006–2010 period (Figure 2B). There were no statistically significant differences between the treatment effectiveness of CD and UC in most eras.

Among patients with CD, the effective ratio of methotrexate monotherapy increased, whereas patients with UC showed a decreasing trend, except in the period after 2016 (Supplementary Figure S1). We then incorporated the treatment era along with other possible confounding factors into the multivariable logistic regression analysis (Table 2). The results demonstrated that there were no statistically significant differences in methotrexate effectiveness between CD and UC (OR: 1.359, 95% CI: 0.988–1.863, *p* = 0.058).

**TABLE 2:** Multivariable analysis of factors affecting methotrexate monotherapy effectiveness.

Factor	Hazard ratio	95% CI	*p* Value
Diagnosis			
UC	1.359	0.988–1.863	
CD	reference		0.058
Therapy period	1.262	1.089–1.467	**0.002**
Time from diagnosis to methotrexate initiation	1.033	1.017–1.049	**<0.001**
Gender			**0.023**
Female	1.395	1.049–1.861	
Male	Reference		
Age at diagnosis	1.038	1.027–1.050	**<0.001**
Smoking history			0.131
Yes	0.795	0.589–1.069	
No	Reference		

Significant *p* values shown in bold; CD, Crohn’s disease; UC, ulcerative colitis; CI, confidence interval.

### Clinical characteristics and methotrexate monotherapy effectiveness

For patients with CD, multivariable analysis showed that after controlling for confounding factors, methotrexate monotherapy appeared to be more effective in older patients (OR: 1.028 per year; 95% CI: 1.017–1.040; *p* < 0.001). In addition, the more recent the treatment, the better the treatment outcomes with methotrexate (OR: 1.363, 95% CI: 1.152–1.623, *p* = 0.001). Patients with exclusive upper gastrointestinal tract disease were considered to receive more effective treatment than those with ileo-colon involvement (OR: 3.797, 95% CI: 0.973–14.855, *p* = 0.049). Univariate logistic regression showed statistical significance for gender, disease behaviour, and perianal involvement; however, no statistical significance was obtained with multivariable logistic regression. No correlation was found between smoking history and the time from diagnosis to methotrexate initiation (Table 3).

**TABLE 3:** Univariate and multivariable logistic regression analyses were performed to identify factors affecting the effectiveness of methotrexate monotherapy in patients with CD and UC.

Factor	CD				UC			
	Univariable		Multivariable		Univariable		Multivariable	
	OR and 95%CI	*p* Value	OR and 95% CI	*p* Value	OR and 95%CI	*p* Value	OR and 95% CI	*p* Value
Age at diagnosis	1.036 (1.025–1.047)	**<0.001**	1.028 (1.017–1.040)	**<0.001**	1.013 (0.995–1.033)	**0.168**	1.009 (0.989–1.029)	0.391
Gender		**0.050**		0.143		**0.140**		
Female	1.384 (1.002–1.922)		1.293 (0.919–1.829)		1.477 (0.881–2.488)			
Male	Reference		Reference		Reference			
Smoking history		0.909				**0.076**		0.164
Yes	0.981 (0.709–1.353)				1.613 (0.951–2.737)		1.492 (0.848–2.625)	
No	Reference				Reference		Reference	
Therapy period	1.550 (1.318–1.833)	**<0.001**	1.363 (1.152–1.623)	**0.001**	1.224 (0934–1.626)	**0.152**	1.213 (0.923–1.614)	0.173
CD location								
Ileal	1.454 (0.988–2.146)	**0.058**	0.991 (0.652–1.505)	0.965				
Colonic	1.487 (1.000–2.216)	**0.050**	1.232 (0.806–1.883)	0.334				
Exclusive upper GI Crohn’s	3.587 (0.970–13.275)	**0.049**	3.797 (0.973–14.855)	**0.049**				
Ileo-colonic	Reference		Reference					
UC location								
Procticis (E1)					1.142 (0.436–2.892)	0.781		
Left sided (E2)					0.825 (0.479–1.421)	0.487		
Extensive (E3)					Reference			
Behaviour								
Stenosing	0.665 (0.457–0.955)	**0.030**	0.863 (0.579–1.274)	0.462				
Internal penetrating	0.431 (0.210–0.813)	**0.014**	0.709 (0.333–1.402)	0.345				
Inflammatory and other	Reference		Reference					
Perianal involvement		**0.002**		0.245				
Yes	0.540 (0.364–0.787)		0.781 (0.511–1.178)					
No	Reference		Reference					
Time from diagnosis to methotrexate initiation	1.005 (0.990–1.020)	0.482		1.041 (1.011–1.071)	**0.006**	1.056 (1.021–1.092)	**0.002**	

Significant *p* values shown in bold; CD, Crohn’s disease; UC, ulcerative colitis; OR, odds ratio; CI, confidence interval; GI, gastrointestinal tract.

For patients with UC, univariate logistic regression showed statistical significance for age, gender, smoking history, treatment eras, and time from diagnosis to methotrexate initiation; after controlling for confounding factors, the multivariable analysis only demonstrated statistical significance for the time interval from diagnosis to methotrexate initiation (Table 3).

### Duration of effective treatment

For patients in whom methotrexate monotherapy was deemed effective (301), 68.8% (207) were still treated with methotrexate at the time of data lock. The Kaplan-Meier curve illustrated that over 50% of this group was still on methotrexate at data lock in both the CD and UC groups. (Figure 2C, *p* = 0.56, log-rank test).

Of the patients treated with methotrexate monotherapy, 532 (51.1%) required treatment escalation after a median time of 5 years in the CD group, 11 years in the UC group, and earlier escalation in the CD group. (Figure 2D; *p* < 0.0001 for the log-rank test).

Of the patients treated with methotrexate monotherapy, at 1 and 3 years, 78.3% and 55.9% of patients with CD and 85.7% and 69.5% of patients with UC, respectively, remained on methotrexate and did not require treatment escalation to biologics or surgery (Supplementary Table S1).

### Development and validation of prognostic nomograms for predicting surgery in CD patients treated initially with methotrexate monotherapy

**FIGURE 3:**
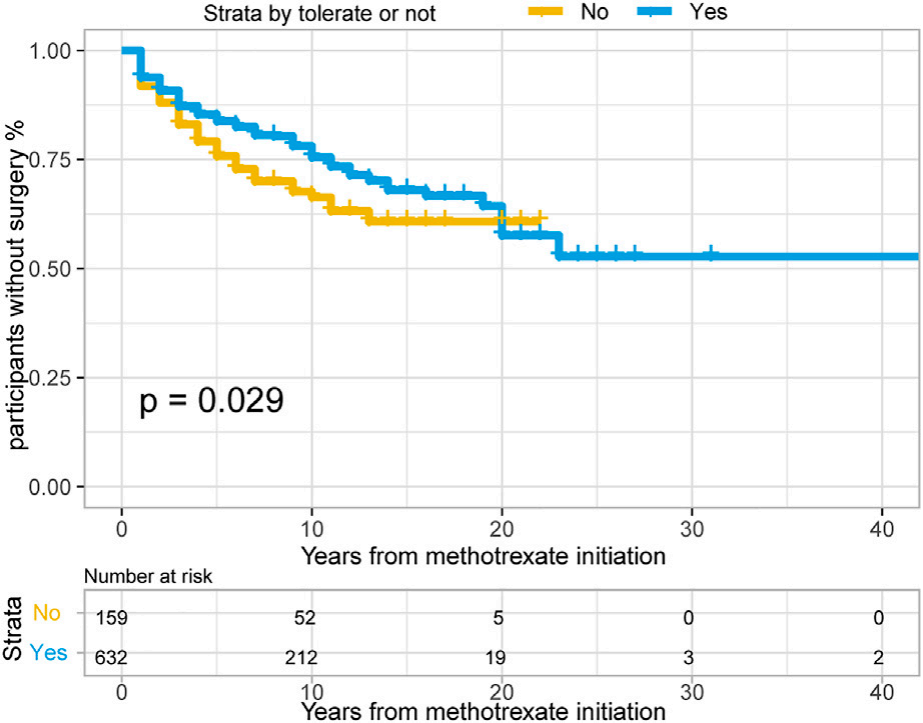
The Kaplan-Meier curve shows time interval from methotrexate initiation to surgery occurance between “Able to tolerate” and “Unable to tolerate” groups of CD patients. (*p* = 0.029 for log-rank test).

In total, 382/791 patients with CD (48.3%) treated with methotrexate monotherapy underwent surgery, 202/791 (25.5%) patients underwent at least two surgeries, and 198/791 (25.0%) underwent surgery after methotrexate initiation. The rates of 1-, 3- and 5- years no-surgery were 93.4%, 86.4% and 82.1%, respectively. A total of 159 patients with CD could not tolerate treatment, and their treatment time interval to surgery was modestly shorter than that of the 632 methotrexate-tolerant patients (Figure 3; *p* = 0.029 for the log-rank test).

**FIGURE 4:**
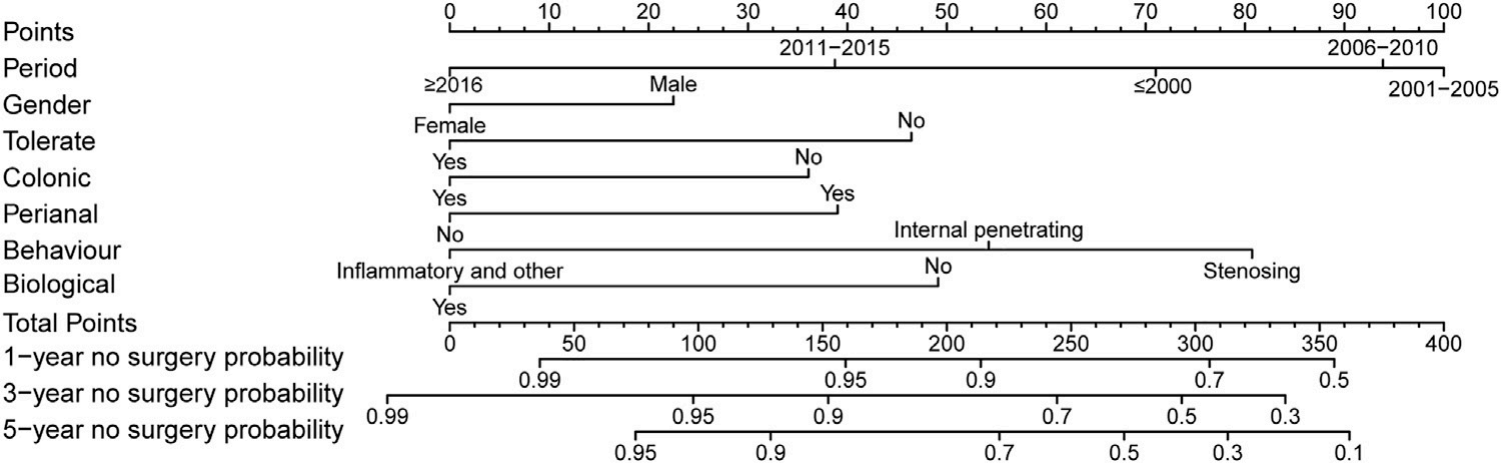
Nomogram for 1-, 3- and 5- years no surgery probability in patients with CD treated with methotrexate monotherapy. Find the point for each variable, sum the scores achieved for each covariate, and locate this sum on the “Total Points” axis. Draw a straight line to determine the likelihood of no surgery probability of 1, 3 or 5 years. (Period: treatment era stratified by 5 years; Tolerate: if or not can tolerate methotrexate; Colonic: disease location; Perianal: have perianal involvement or not; Behaviour: disease characteristic: stenosing, internal penetrating or inflammatory and others; Biological: biological therapy requirment).

**FIGURE 5:**
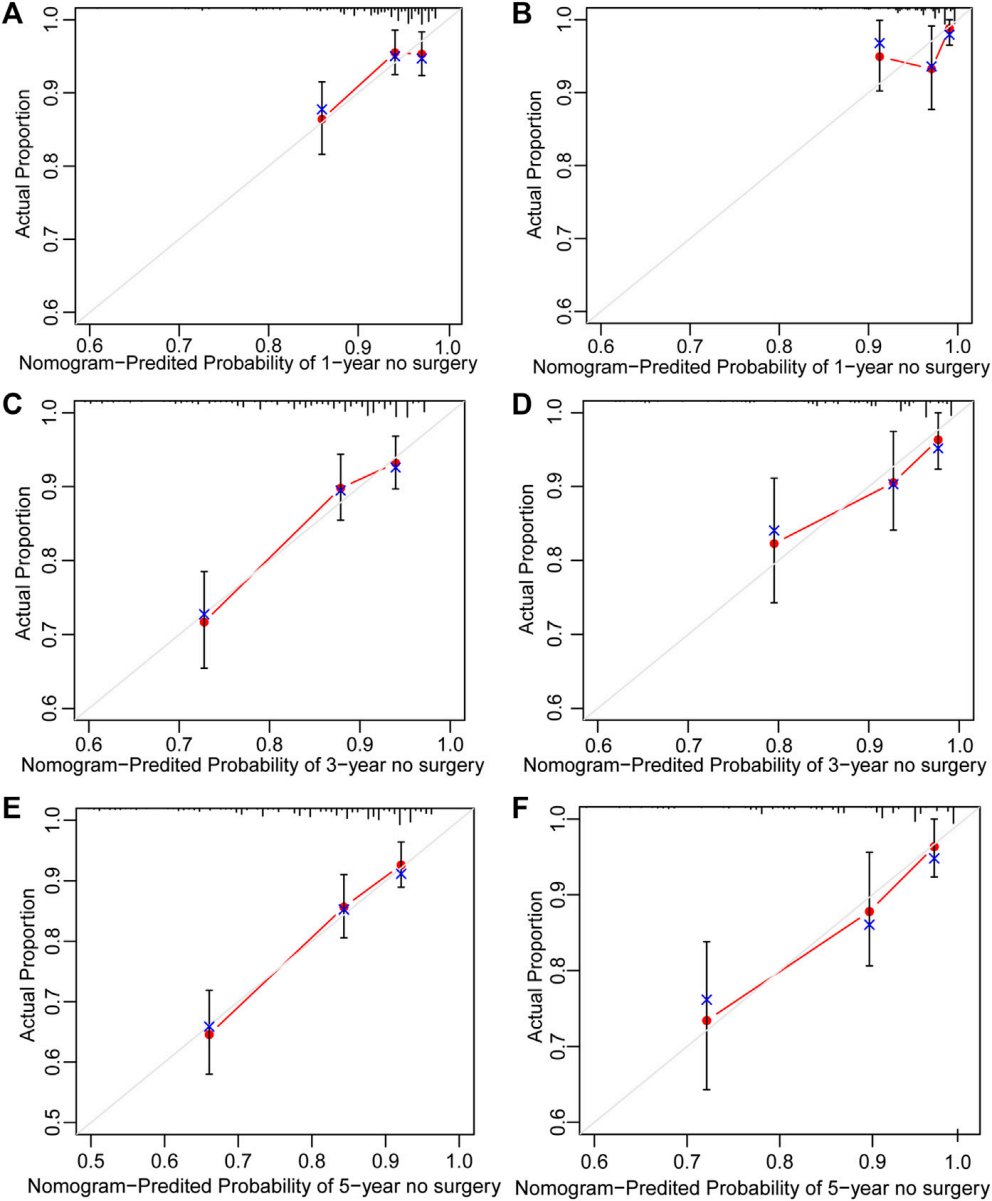
Calibration curve of nomogram for predicting no surgery occurrence at **(A)** 1-, **(C)** 3-, and **(E)** 5- years in the training cohort, and at **(B)** 1-, **(D)** 3-, and **(F)** 5- years in the validation cohort. The actual proportion is plotted on the Y-axis and the nomogram-predicted probability is plotted on the X-axis. (CD, Crohn’s disease).

Surgery occurrence and time interval in the treatment process are important indicators of treatment effectiveness. A total of 791 patients with CD were randomly divided into training and validation cohorts at a ratio of 7:3. There was no statistically significant difference between the two groups (Supplementary Table S2). Finally, the variables included in the nomogram were sex, disease location, disease behaviour, perianal involvement, tolerance, treatment period, and biological requirements based on outcomes of Cox regression (Table 4; Figure 4). The C-index was 0.711 [0.664–0.758] in the training cohort and 0.732 [0.661–0.803] in the validation cohort. As shown in Figure 5, the calibration curves showed good agreement between predictions of 1-, 3-, and 5- year of surgery occurrence and the actual observations in both the training and validation cohorts.

**TABLE 4:** Univariate and multivariable Cox regression for determining factors affecting surgery in patients with CD and UC treated with methotrexate monotherapy.

Factor	CD				UC			
	Univariable		Multivariable (backward stepwise)		Univariable		Multivariable (backward stepwise)	
	OR and 95% CI	*p* Value	OR and 95% CI	*p* Value	OR and 95% CI	*p* Value	OR and 95% CI	*p* Value
Age at diagnosis	0.981 (0.969–0.992)	**<0.001**			0.940 (0.885–0.998)	**0.042**	0.936 (0.880–0.996)	**0.038**
Gender		**0.019**		**0.040**		**0.083**		0.070
Female	0.681 (0.494–0.938)		0.710 (0.512–0.985)		0.156 (0.019–1.274)		0.143 (0.018–1.172)	
Male	Reference		Reference		Reference		Reference	
Smoking history		0.638				0.472		
Yes	0.924 (0.666–1.283)				0.555 (0.112–2.759)			
No	Reference				Reference			
Therapy period	0.769 (0.663–0.892)	**<0.001**	0.810 (0.695–0.943)	**0.007**	0.928 (0.449–1.920)	0.841		
Disease location								
Ileal	0.740 (0.509–1.075)	0.114	0.935 (0.624–1.401)	0.745				
Colonic	0.418 (0.271–0.645)	**<0.001**	0.537 (0.342–0.841)	**0.007**	<0.001 (0-Infinity)	0.999		
Exclusive upper GI Crohn’s	1.001 (0315–3.181)	0.998	1.510 (0.462–4.936)	0.495	0.518 (0.124–2.172)	0.368		
Ileo-colonic	Reference		Reference		Reference			
Behaviour								
Stenosing	2.137 (1.495–3.053)	**<0.001**	1.843 (1.272–2.671)	**0.001**				
Internal penetrating	3.815 (2.453–5.933)	**<0.001**	2.775 (1.734–4.441)	**<0.001**				
Inflammatory and other	Reference		Reference					
Perianal involvement		**<0.001**		**0.009**				
Yes	1.923 (1.391–2.659)		1.609 (1.125–2.299)					
No	Reference		Reference					
Time from diagnosis to methotrexate initiation	0.991 (0.974–1.008)	0.286		1.000 (0.918–1.088)	0.991			
Tolerate		**0.074**		**0.004**		**0.079**		
Yes	0.716 (0.497–1.032)		0.560 (0.379–0.828)		0.238 (0.048–1.179)			
No	Reference		Reference		Reference			
Previous surgery		**0.043**						
Yes	1.403 (1.011–1.947)							
No	Reference							
Biologics requirement		**0.151**		**0.004**		0.664		
Yes	0.790 (0.572–1.090)		0.605 (0.430–0.852)		1.36 (0.340–5.438)			
No	Reference		Reference		Reference			
Glucocorticoid requirment		0.459				0.998		
Yes	1.191 (0.750–1.892)				<0.001 (0-Infinity)			
No	Reference				Reference			

Significant *p* values shown in bold; CD, Crohn’s disease; UC, ulcerative colitis; OR, odds ratio; CI, confidence interval; GI, gastrointestinal tract.

### Establishment and validation of prognostic nomograms for predicting surgery in UC patients treated initially with methotrexate monotherapy

**FIGURE 6:**
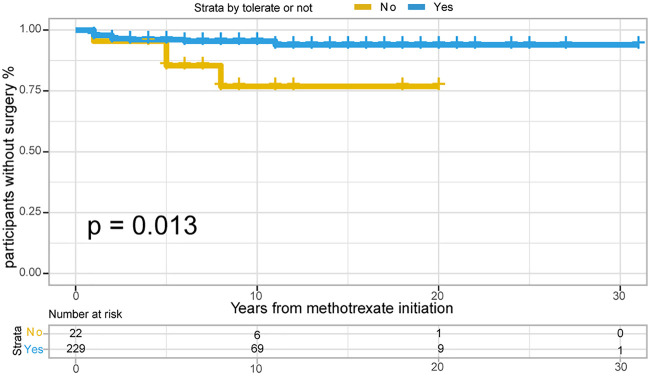
The Kaplan-Meier curve shows time interval from methotrexate initiation to surgery occurance between “Able to tolerate” and “Unable to tolerate” groups of UC patients. (*p* = 0.013 for log-rank test).

Of the 251 patients with UC, 15 (6.0%) underwent colectomy. The indications for colectomy included acute severe colitis in 6 patients (40%), chronic continuous colitis in 6 patients (40%), dysplasia in 1 patient (6.7%), and undocumented reasons in 2 patients. The 1-, 3- and 5- years no-surgery rates were 97.6%, 96.0%, and 95.1%, respectively. Overall, 4/22 (18.2%) patients who were unable to tolerate methotrexate required colectomy, compared with 11/229 (4.8%) patients who could tolerate methotrexate. Time to colectomy was shorter in individuals unable to tolerate methotrexate than in those able to tolerate methotrexate (Figure 6, *p* = 0.013 for log-rank test).

**FIGURE 7:**
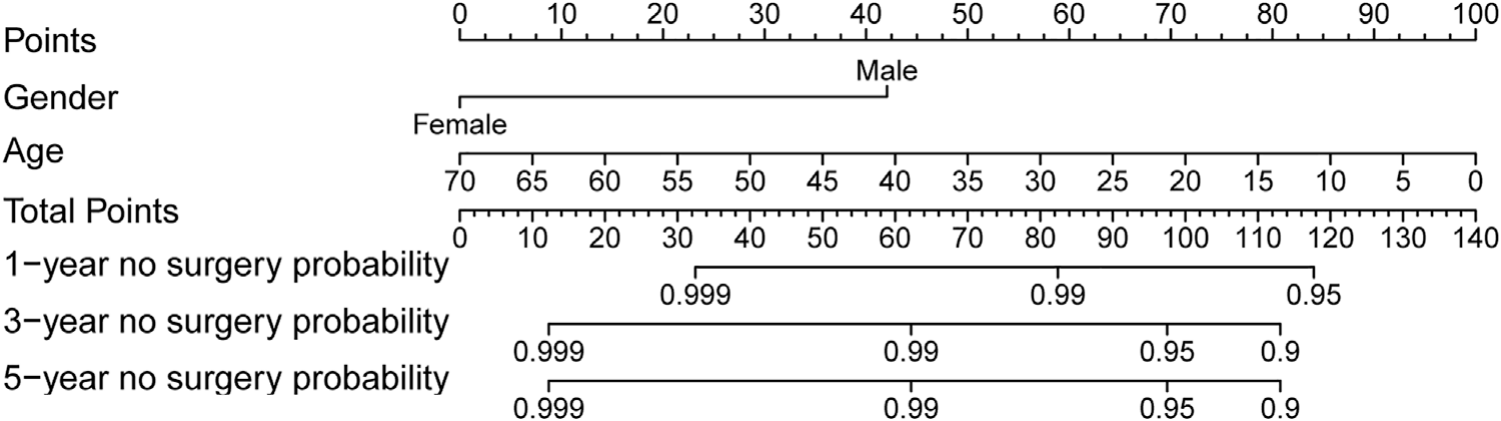
Nomogram for 1-, 3- and 5- years no surgery probability in UC patients treated with methotrexate monotherapy. Find the point for each variable, sum the scores achieved for each covariate, and locate this sum on the “Total Points” axis. Draw a straight line to determine the likelihood of no surgery probability of 1, 3 or 5 years. (Age: age at UC diagnosis).

**FIGURE 8:**
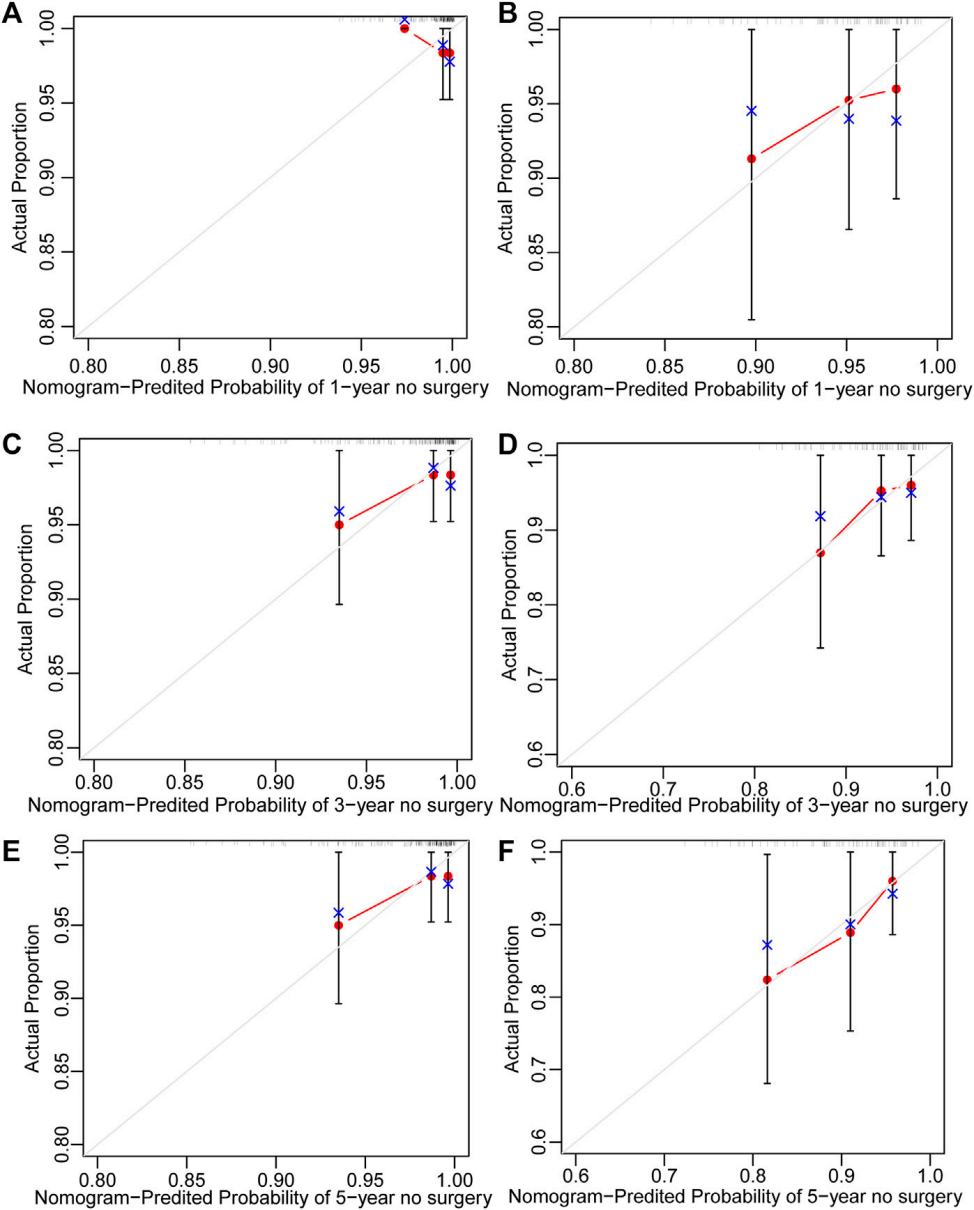
Calibration curve of nomogram for predicting no surgery occurrence at **(A)** 1-, **(C)** 3-, and **(E)** 5-years in the training cohort, and at **(B)** 1-, **(D)** 3-, and **(F)** 5- years in the validation cohort. The actual proportion is plotted on the Y-axis and the nomogram-predicted probability is plotted on the X-axis. (UC: ulcerative colitis).

251 UC patients with were randomly divided into training and validation cohorts at a ratio of 7:3. There was no statistically significant difference between the two groups (Supplementary Table S3). All factors were analysed using univariate regression analysis to identify potential risk variables. Potential risk factors (*p* < 0.2 in univariate regression analysis) were selected for stepwise regression analysis with backward selection procession by AIC. Finally, the variables included in the nomogram were sex and age at UC diagnosis (Table 4; Figure 7). The C-index was 0.784 [0.600–0.968] and 0.690 [0.502–0.878] in the training and validation cohorts, respectively. As shown in Figure 8, the calibration curves showed good agreement between the predictions of surgery occurrence and the actual observations in both the training and validation cohorts.

## Discussion

Through this retrospective study that enrolled 791 patients with CD and 251 with UC, we report the first large-scale study assessing the clinical characteristics and patient-level outcomes of patients with CD and UC treated with methotrexate, using the IBD BioResource. Currently, non-biological treatments remain valuable approaches for patients with IBD; however, their long-term outcome data remain sparse. In our study, 8.25% of patients had been treated with methotrexate, either as a monotherapy or combination therapy, and methotrexate provided an effective long-term treatment for 35.5% of patients with UC and 26.7% with CD without escalation therapy. A multicentre retrospective study in the Netherlands suggested that 86%, 63%, 47%, and 20% of patients with CD who were treated with methotrexate monotherapy obtained clinical benefits at 6, 12, 24, and 60 months, respectively (Seinen et al., 2013). Our study also evaluated the tolerability and safety of methotrexate; of the 1042 patients, 181 (17.4%) patients were unable to tolerate and had to stop methotrexate, consistent with previous studies (Herfarth et al., 2016). Nausea was the most common adverse reaction, especially in the intolerance group; hepatotoxicity and fatigue were also observed without fatal adverse reactions. A retrospective study published in 2022 reported that methotrexate-induced nausea was noted in 34% of paediatric patients with IBD (Mehta et al., 2022). In our study, over 50% of methotrexate-effective patients were still on treatment at data lock in the two groups. Methotrexate treatment is relatively safe, with a low rate of adverse reactions and no fatal adverse reactions.

In this study, methotrexate appeared effective in both CD and UC, with no difference, consistent with a previous cohort study (Wahed et al., 2009). The ECCO Guidelines recommend methotrexate for the maintenance of remission in patients with steroid-dependent CD, however, for patients with UC, no clear application guidelines are available. In our study, the CMH test was more effective in UC than in CD; we also found evidence of increased methotrexate effectiveness in CD and a decreasing trend in UC (Supplementary Figure S1). Multivariable logistic regression analysis showed no statistical significance between CD and UC (*p* = 0.058) in the adjusted models. By further comparison, methotrexate had a better effect on patients with UC than on those with CD in avoiding escalation treatment (Figure 2D). Surgical risk factors include perianal involvement, internal penetration, and stenosis. After excluding these patients, the Kaplan-Meier curve showed that the protective effect of methotrexate on CD was significantly increased, and the gap between CD and UC was decreased (Supplementary Figure S2 vs. Figure 2D). We excluded patients with UC who had undergone colectomy, which resulted in a bias for milder disease among the participants. Those who had undergone CD-related surgery were analysed, showing a bias in disease severity. Our study is based in a single region of the United Kingdom with a limited number of cases, and endoscopy estimation was not available. In the future, prospective RCTs are needed to compare the efficacy of methotrexate in CD and UC.

A previous review considered that maintenance methotrexate treatment provides acceptable remission rates for treatment periods up to 3 years, after which relapse is frequent and occurs early (usually within 1 year) (Fraser et al., 2002). In the current study, over 50% of the patients in whom treatment was deemed effective were still on methotrexate at the data lock, with a median treatment duration of 5 (CD) and 11 (UC) years. It seems that methotrexate is adhered to for prolonged periods (and effective), despite well-documented concerns about increased risk of hepatotoxicity and long-term blood cell reduction.

Our analysis identified specific patient subgroups that were most likely to respond to methotrexate. These included older patients treated in the recent period with exclusive upper GI disease for CD. Older patients with better outcomes may have a milder course (Charpentier et al., 2014). Although the *p* value of upper GI disease was less than 0.05, the OR value contains 1, and previous studies have shown that upper GI disease (*p* = 0.042) was significantly associated with an increased risk of surgical recurrence (Frolkis et al., 2013), larger trials are needed to confirm this. For UC, in multivariable analysis, the time interval from diagnosis to methotrexate initiation was an independent factor associated with effectiveness of treatment (OR: 1.056, 95% CI: 1.021–1.092 per year). After further analysis, we found that the methotrexate-tolerant group had a longer median time interval between diagnosis and methotrexate initiation than the methotrexate-intolerant group (5 years vs. 3 years).

We further analysed the patients’ clinical characteristics and occurrence of surgery. Identification of patients at the highest risk of disease progression, who have the greatest chance of benefiting from early initiation of effective therapy, is an aspirational goal. For CD, 202/791 (25.5%) patients underwent at least two surgeries, and 198/791 (25.0%) underwent surgery after methotrexate initiation. We identified factors associated with surgery occurrence through univariate Cox regression as follows: age at diagnosis, gender, therapy era, disease location, perianal involvement, disease behaviour, tolerance, previous surgery history, biologics, and glucocorticoid treatment. For UC, 15/251 (6.0%) patients required colectomy after methotrexate treatment. Univariate Cox regression analysis revealed that the age at diagnosis, sex, and tolerance were significantly associated with early surgery. We then performed stepwise regression based on AIC to select factors into the final prognostic model so that clinicians could identify patients at higher risk of surgery and implement more aggressive medical management as required.

Surgery is generally considered as a last-resort treatment for IBD. A review published in 2013 suggested that the 1- and 5- year surgery risk after diagnosis of UC was 4.9% and 11.6%, respectively, and that of CD was 16.3% and 33.3%, respectively (Samuel et al., 2013a). For patients diagnosed in the 21st century, the cumulative 5-year risk of surgery was 7.0% for UC and 17.8% for CD, substantially lower than the previous review (Tsai et al., 2021). In our study, for CD, the rates of 1- and 5- year surgery occurrence were 6.6% and 17.9%, respectively. For UC, the rates of 1- and 5- year surgery occurrence were 2.4% and 4.9%, respectively. In our study, the surgery rates were lower than those reported, reflecting the effectiveness of methotrexate. Although the number of surgeries has been decreasing, it still poses a huge burden to patients. One of the biggest challenges clinicians face today is predicting which patients are prone to surgery requirements. Those at the highest risk of surgery may benefit the most from early surgery.

As the nomograms show, female is a predictor of a lower risk of surgical treatment for both, patients with CD and UC. A consensus has been reached that male sex may be associated with a greater risk of growth impairment, and growth impairment diagnosis predicts an increased risk of bowel surgery (Ricciuto et al., 2021). A previous study of UC showed that male sex was significantly associated with colectomy risk (Samuel et al., 2013b; Macaluso et al., 2019). Jia-Yin et al. (Yao et al., 2020) developed a nomogram to predict 1-year surgery risk in patients with CD without the sex variable. The people we enrolled were all from the United Kingdom, therefore, regional bias should be accounted for.

Smoking cessation is generally advised for patients with CD. However, in our study, smoking history was not a predictor of surgery occurrence in either UC or CD. Current smokers had a reduced risk of UC (odds ratio [OR]:0.61) and an increased risk of developing CD (OR: 1.74) (García Rodríguez et al., 2005). However, most studies showed that smoking status was not associated with surgical requirements (Hoie et al., 2007; Solberg et al., 2014). However, we could not confirm this association in the present study.

Regarding disease location, consistent with a previous study (Ricciuto et al., 2021), isolated colonic disease was associated with fewer surgeries. In our study, the colonic type had a lower surgery risk than the ileo-colonic type (OR: 0.537, 95% CI: 0.342–0.841), and was included in the nomogram as a predictor. For patients with UC, extensive disease is viewed as a risk factor for colectomy. Extensive colonic disease can be considered as a manifestation of severe disease (Hoie et al., 2007; Macaluso et al., 2019). In our study, the long-term efficacy of methotrexate for UC was independent of lesion location. Consistent with a previous study (Peyrin-Biroulet et al., 2010; Thia et al., 2010; Peyrin-Biroulet et al., 2012; Solberg et al., 2014; Yao et al., 2020), perianal involvement and behaviour were included in the nomogram as predictors of the occurrence of surgery. A review of 70 studies concluded that a younger age at diagnosis was associated with a higher risk of colectomy (Macaluso et al., 2019). Further analysis showed that colonic disease accounted for 28.5% (112/393) of patients below 30 years of age and 30.2% (120/398) of patients above 30 years of age. However, we believe that this is not enough to cause the difference and speculate that the risk of surgery is higher in young patients due to their own disease characteristics. Patients who received biologics after methotrexate initiation had a lower risk of surgery (OR: 0.605, 95% CI: 0.430–0.852). This suggests that for patients who cannot be controlled by traditional drugs or who do not respond to drugs, biologic agents should be sought in time to reduce the risk of surgery.

It is noteworthy that the value of surgery for IBD is changing (Buskens and Bemelman, 2019). We need to be aware that surgery should be considered as an alternative treatment rather than a salvage procedure after failed conservative treatment. We hope to use the nomograms to identify the high risk of surgery and administer more aggressive treatments to achieve better outcomes.

## Limitations

Our study had some limitations. In our study, the effectiveness of treatment was judged according to patients’ subjective perception and whether there is treatment escalation. There is no standard disease activity score to evaluate the efficacy. Our study was based on IBD BioResource, a United Kingdom hospital-based program, and hence there may be regional limitations and disease severity bias, which may include individuals with more severe disease. Our results are based on a large sample, so we believe that this study is of certain significance.

## Conclusion

In conclusion, methotrexate was effective in both, patients with CD and UC, with no significant difference between two groups. Methotrexate is well tolerated by most individuals. Male sex, methotrexate intolerance, and perianal involvement are predictors of early surgery. Treatment in the modern era, colonic and inflammatory diseases, and biological requirements are predictors of low surgery occurrence for CD. Male sex and younger age at diagnosis are predictors of early surgery in patients with UC. The nomograms can predict the 1-, 3- and 5- year risk of surgery for patients with CD and UC, who were initially treated with methotrexate monotherapy, with accuracy and discriminative ability.

## Data Availability

Publicly available datasets were analyzed in this study. This data can be found here: We obtained the data from the United Kingdom IBD BioResource (www.ibdbioresource.nihr.ac.uk), which launched in 2016 as part of the United Kingdom National Institute for Health Research BioResource. To obtain these data, a data applycation was submitted to IBD BioResource (https://www.ibdbioresource.nihr.ac.uk/index.php/resources/applying-for-access-to-the-ibd-bioresource-panel-2/).

## Abbreviation

IBD, inflammatory bowel disease; CD, Crohn’s disease; UC, ulcerative colitis; IBDU, IBD-unclassified; RCT, randomised control trial; ECCO, The European Crohn’s and Colitis Organisation; OR, odds ratio; CI, confidence interval; GI, gastrointestinal tract.
