# Supplementary material for: Methotrexate showed efficacy both in Crohn’s disease and ulcerative colitis, predictors of surgery were identified in patients initially treated with methotrexate monotherapy
Source: Front Pharmacol. 2022 Sep 26;13:996065. doi: 10.3389/fphar.2022.996065 (PMC9548616; doi:10.3389/fphar.2022.996065)

Supplementary Figure 1. The proportion of CD and UC patients who considered methotrexate monotherapy effective was stratified by treatment period. (CD, Crohn's disease; UC, ulcerative colitis)

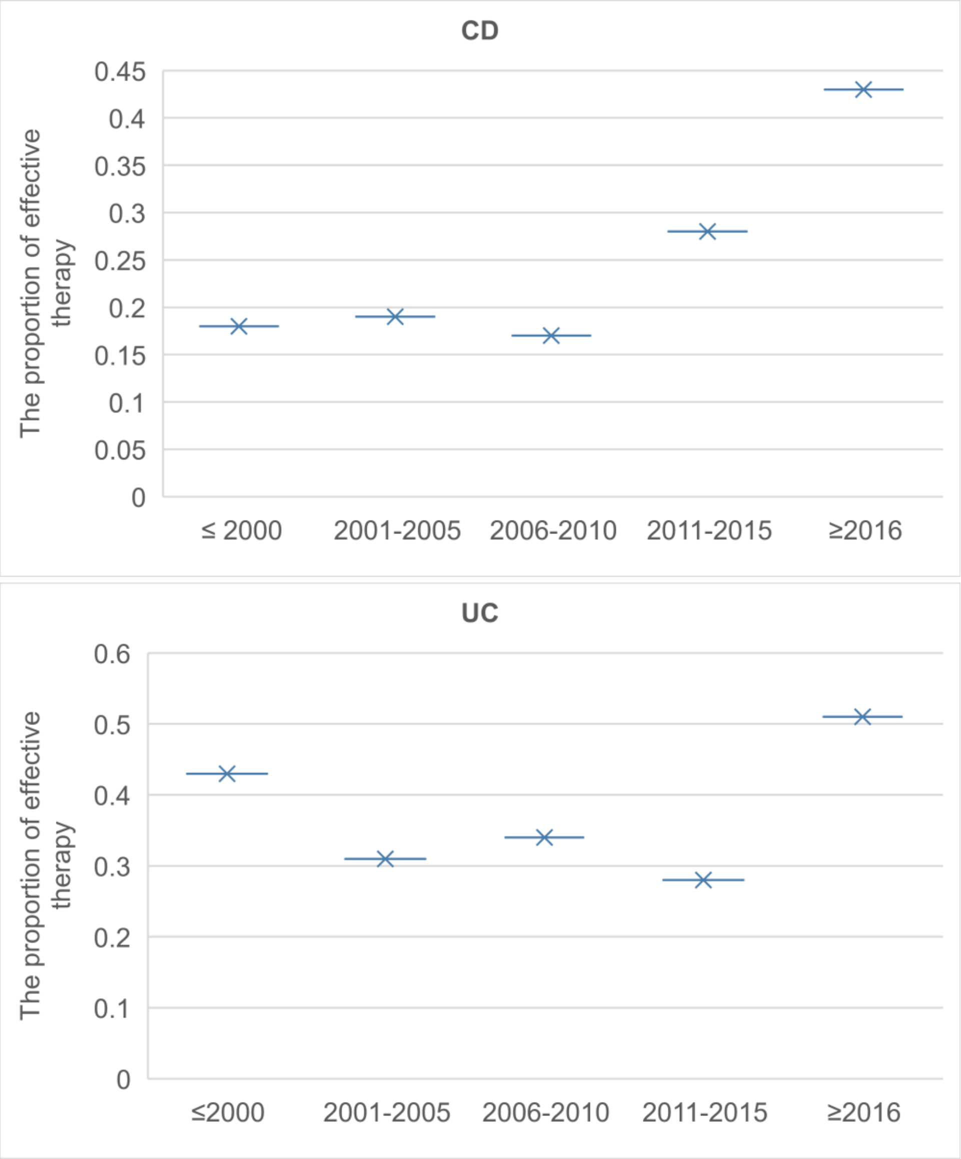

Supplementary Table 1. The percentage of participants who remained on methotrexate and did not require treatment escalation to biological therapy or surgery.

		CD	UC
All patients		791	251
Median time without escalationg and 95%CI (year)		5 (4-5)	11 (8-17)
Percent of patients without treatment escalation after N years methotrexate initiation	1	78.3%	85.7%
	3	55.9%	69.5%
	5	45.1%	62.0%
	10	32.2%	51.6%

CD, Crohn's disease; UC, ulcerative colitis; CI, confidence interval.

Suplementary Table 2. Demographic and clinical characteristics in the training and validation CD cohort.

**Factor**	Training cohort (N=554)	Validating cohort (N=237)	*P* value
**Age at diagnosis, median (IQR)**	29.0 (20.0, 46.0)	30.0 (21.0, 45.0)	0.74
**Time from diagnosis to methotrexate initiation**	5.0 (1.0, 13.0)	6.0 (2.0, 14.0)	0.46
**Gender**			0.21
Male	223 (40.3%)	107 (45.1%)	
Female	331 (59.7%)	130 (54.9%)	
**Smoking history**			0.23
No	329 (59.4%)	152 (64.1%)	
Yes	225 (40.6%)	85 (35.9%)	
**Disease location**			0.52
Ileal	175 (31.6%)	85 (35.9%)	
Colonic	161 (29.1%)	71 (30.0%)	
Ileo-colonic	210 (37.9%)	79 (33.3%)	
Exclusive upper GI Crohn’s	8 (1.4%)	2 (0.8%)	
**Perianal involvement**			0.54
Yes	158 (28.5%)	62 (26.2%)	
No	396 (71.5%)	175 (73.8%)	
**Behaviour**			0.44
Stenosing	160 (28.9%)	64 (27.0%)	
Internal penetrating	53 (9.6%)	17 (7.2%)	
Inflammatory and others	341 (61.6%)	156 (65.8%)	
**Theray period**			0.56
≤2000	29 (5.2%)	10 (4.2%)	
2001-2005	51 (9.2%)	23 (9.7%)	
2006-2010	167 (30.1%)	59 (24.9%)	
2011-2015	191 (34.5%)	92 (38.8%)	
≥2016	116 (20.9%)	53 (22.4%)	
**Tolerate**			0.63
Yes	440 (79.4%)	192 (81.0%)	
No	114 (20.6%)	45 (19.0%)	
**Previous surgery**			0.08
Yes	366 (66.1%)	65 (27.4%)	
No	366 (66.1%)	172 (72.6%)	
**Biologics requirement**			0.48
Yes	290 (52.3%)	117 (49.4%)	
No	264 (47.7%)	120 (50.6%)	
**Glucocorticoid requirment**			0.63

Yes	65 (11.7%)	31 (13.1%)
No	489 (88.3%)	206 (86.9%)

CD, Crohn's disease; GI, gastrointestinal tract; IQR, interquartile ranges;

Supplementary Table 3. Demographic and clinical characteristics in the training and validation UC cohort.

**Factor**	Training cohort (N=182)	Validation cohort (N=69)	*P* value
**Age at diagnosis, median (IQR)**	38.5 (27.0, 50.0)	36.0 (25.0, 48.0)	0.74
**Time from diagnosis to methotrexate initiation**	5(2, 12)	4(2,12)	0.72
**Gender**			0.48
Male	97 (53.3%)	33 (47.8%)	
Female	85 (46.7%)	36 (52.2%)	
**Smoking history**			0.77
Yes	71 (39.0%)	25 (36.2%)	
No	111 (61.0%)	44 (63.8%)	
**Disease location**			0.81
Procticis	16 (8.8%)	6 (8.7%)	
Left sided	87 (47.8%)	36 (52.2%)	
Extensive	79 (43.4%)	27 (39.1%)	
**Theray period**			0.38
≤2000	5 (2.7%)	2 (2.9%)	
2001-2005	12 (6.6%)	4 (5.8%)	
2006-2010	35 (19.2%)	15 (21.7%)	
2011-2015	77 (42.3%)	36 (52.2%)	
≥2016	53 (29.1%)	12 (17.4%)	
**Tolerate**			0.14
Yes	169 (92.9%)	60 (87.0%)	
No	13 (7.1%)	9 (13.0%)	
**Biologics requirement**			0.67
Yes	77 (42.3%)	27 (39.1%)	
No	105 (57.7%)	42 (60.9%)	
**Glucocorticoid requirment**			1.00
Yes	29 (15.9%)	11 (15.9%)	
No	153 (84.1%)	58 (84.1%)	

IQR, interquartile ranges; UC, ulcerative colitis.

Supplement Figure 2. The Kaplan–Meier curve shows time interval from methotrexate initiation to surgery occurance between UC and CD (excluding patients with perianal involvement, internal penetrating and stenosis) patients. (*P* = 0.046 for log-rank test). (CD, Crohn's disease; UC, ulcerative colitis)

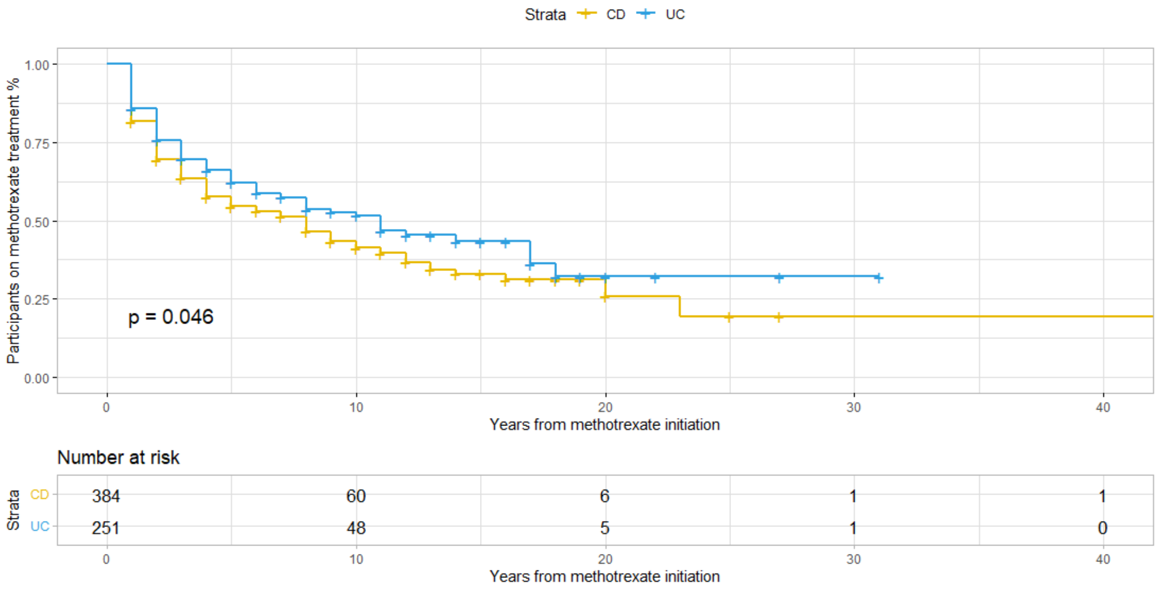

Supplement: Supplementary file 1 [file DataSheet1.PDF]
